# The tree of life of copper-containing amine oxidases

**DOI:** 10.3389/fpls.2025.1544527

**Published:** 2025-04-24

**Authors:** Zaibao Zhang, Tao Xiong, Kejia Li, Kexin Huang, Siyu Wu, Luhui Wu

**Affiliations:** ^1^ School of Life and Health Science, Huzhou College, Huzhou, Zhejiang, China; ^2^ College of Life Sciences, Xinyang Normal University, Xinyang, Henan, China

**Keywords:** CuAO, molecular evolution, origin, diversification, gene duplication

## Abstract

Copper-containing amine oxidases (CuAOs) catalyze the terminal oxidation of polyamines (PAs), producing ammonium, an aminoaldehyde, and hydrogen peroxide (H_2_O_2_). Plant CuAOs are induced by stress-related hormones such as methyl-jasmonate (MeJA), abscisic acid (ABA), and salicylic acid (SA). Mammalian copper-containing amine oxidases (CAOs), encoded by four genes (AOC1-4) that catalyze the oxidation of primary amines to aldehydes, regulate various biological processes and are linked to diseases like inflammatory conditions and histamine intolerance. To understand the evolutionary history and functional divergence of CuAOs, we conducted phylogenetic and expression analyses of CuAOs in plants and animals. In this study, the *copper amine oxidase* (*CuAO*) genes were identified by HMMER and BLASTP, and verified by CDD/HMM/SMART. Multiple sequence alignment was performed using Muscle5, and the phylogenetic tree was constructed by IQ-TREE2. The syntenic relationship was analyzed by MCScanX and CIRCOS. Meanwhile, the expression data of *Arabidopsis thaliana* and human and other species were integrated for analysis. Here, 950 and 264 CuAO orthologues were identified in 188 plant and 79 animal genomes. Phylogenetic analyses indicate that CuAO originated in the common ancestor before the divergence of plants and animals. The copy numbers of CuAOs vary significantly across plant species, whereas they remain relatively stable in animal species, generally maintaining 3-4 copies per species. During the evolutionary process, plant CuAOs formed two clades (I and II), while animal CuAOs formed three clades (CAO-like, AOC1, AOC2-4). Interestingly, plant clade I CuAOs lacks the active site motif T/S-X_1_-X_2_-N-Y-D. The further differentiation of plant clade II CuAOs is related to the preference for X_1_ and X_2_ active sites. CAO-like and AOC1 are monophyletic branches. Mammalian AOC2-4 is separated from non-mammalian AOC2-4, and the differentiation of mammalian AOC3 and AOC4 occurs in a species-specific manner. Our study provides a comprehensive understanding of the evolutionary trajectory of the *CuAO* gene family in plants and animals at the genome-wide level. These findings lay a crucial foundation for future research to conduct in-depth functional characterization.

## Introduction

PAs are a class of small aliphatic nitrogen-containing compounds that are widely present in animals, plants, bacteria, and fungi (Wang et al., 2019). As important regulators of cell growth and differentiation, PAs are involved in basic cellular processes such as DNA replication and transcription, RNA modification, protein synthesis, ion channel regulation, free radical scavenging, cell cycle regulation, signal transduction, and programmed cell death (Tavladoraki et al., 2012; Murray-Stewart et al., 2016). In plants, PAs play crucial roles in flower development and differentiation, leaf development and aging, fruit ripening, and various stress responses (Wang et al., 2019). In mammalian cells, dysregulation of PA metabolism is associated with cancer development, making it an important target for anti-cancer therapy (Boomsma et al., 2003; Dunkel et al., 2008; Salmi and Jalkanen, 2019). The three most common PAs found in plants and animals are putrescine (Put), spermidine (Spd), and spermine (Spm) (Shukla et al., 2020). Their oxidative degradation is mainly catalyzed by copper amine oxidase (CuAO) and polyamine oxidase (PAO). CuAOs mainly catalyze the oxidation of putrescine to produce NH^4+^, 4-aminobutyrate, and H_2_O_2_, with 4-aminobutyrate further converting to γ-aminobutyric acid (Fortes et al., 2019). *CuAOs* not only play roles in plants, but also are widely distributed in mammalian tissues, and their activity changes are related to various diseases, such as histamine intolerance and rheumatism (Boomsma et al., 2003; Dunkel et al., 2008; Salmi and Jalkanen, 2019).

CuAOs are widely occurred enzymes that catalyze the oxidative deamination of primary amines and PAs such as diamines and histamine (Pietrangeli et al., 2003). Their molecular form is a dimer, with subunit molecular weights ranging from 70 to 95 kDa (Tavladoraki et al., 2016; Fraudentali et al., 2021). The enzymatic activity of CuAOs depends on two cofactors: covalently bound 2,4,5-trihydroxyphenylalanine quinone (TPQ) and a copper ion involved in the catalytic cycle (Brazeau et al., 2004). TPQ is produced by the modification of endogenous tyrosine residues, and copper ions play a key role in the biogenesis and catalytic reactions of TPQ-mediated substrate oxidation (Brazeau et al., 2004). Mammalian CuAOs have a highly conserved T/S-X_1_-X_2_-N-Y-D motif at the active site, where tyrosine is post-translationally modified to TPQ (Lopes de Carvalho et al., 2019). CuAOs primarily catalyze the terminal oxidation of diamines (such as Put) and, in some cases, the oxidation of higher PAs (such as Spd), thus playing a crucial role in the early steps of PA oxidative metabolism and regulating specific subcellular compartments of PA levels (Fraudentali et al., 2020a). CuAOs catalytic reactions involve three main processes: (i) TPQ biosynthesis, (ii) amine substrate oxidation to reduce TPQ, and (iii) molecular oxygen reduction via reduced TPQ (Brazeau et al., 2004). All of these reactions require the participation of molecular oxygen (Brazeau et al., 2004).

CuAOs have various biological functions in plants and animals. In *Arabidopsis thaliana*, there are 10 *CuAO* genes, of which *AtCuAOε1* (*AT4G12270*) and *AtCuAOε2* (*AT4G12280*) lack essential active residues and are considered to be fragment copies of *AtCuAOδ* (*AT4G12290*) (Qu et al., 2014; Tavladoraki et al., 2016; Fraudentali et al., 2020a). *AtCuAOα3* (*AT1G31710*) and *AtCuAOζ* (*AT2G42490*) play roles in regulating the stability of PA in peroxisomes (Planas-Portell et al., 2013). *AtCuAOγ1* (*AT1G62810*) primarily involves NO production mediated by PAs and/or abscisic acid (ABA), while *AtCuAOα2* (*AT1G31690*) regulates arginase activity to affect the availability of arginine and thus participates in NO production, revealing a new regulatory pathway for NO production in plants (Tavladoraki et al., 2016; Groß et al., 2017). *AtCuAOδ* plays a significant role in ABA-induced stomatal closure (Fraudentali et al., 2019), while *AtCuAOβ* (*AT4G14940*) is involved in the stomatal regulation process under mechanical damage conditions (Fraudentali et al., 2023). Furthermore, *AtCuAOβ* is involved in both MeJA-induced early root protoxylem differentiation and the response to wounding (Ghuge et al., 2015a; Ghuge et al., 2015b). The expression of *AtCuAOζ* in guard cells indicates that it is involved in ABA-mediated stomatal opening control (Qu et al., 2014). AtCuAOβ, AtCuAOγ1, and AtCuAOγ2 are located in the apoplast (Fraudentali et al., 2020a; Fraudentali et al., 2020b). Additionally, AtCuAOα2 and AtCuAOα3 are located in peroxisomes (Fraudentali et al., 2020a). To date, the only vacuolar located CuAO isoform is AtCuAOδ (Fraudentali et al., 2019).There are 8 *CuAO* genes in *Solanum lycopersicum* genome, among which 6 *CuAOs* (*SlCuAO1, 2, 3, 4, 6, 7*) are specifically expressed in the roots, *SlCuAO5* is specifically expressed in the fruits, and *SlCuAO8* is specifically expressed in the flowers (Upadhyay et al., 2024a). This indicates that the expression of amine oxidases is diverse in different tissues, and it is particularly prominent in the roots (Upadhyay et al., 2024a). In humans, there are four genes that encode *CuAO*: *AOC1* (diamine oxidase), *AOC2* (retinal-specific amine oxidase), *AOC3* (vascular adhesion protein-1, VAP-1), and *AOC4* (pseudogene) (Finney et al., 2014). *AOC1* is mainly expressed in the kidney, placenta, intestine, thymus, and seminal vesicle, and is the main enzyme for metabolizing histamine (Elmore et al., 2002; Maintz and Novak, 2007). Its activity is related to the risk of pregnancy (Maintz et al., 2008). *AOC2* was initially cloned from the retina and is also expressed in adipose tissue, playing a role in the process of adipocyte differentiation (Imamura et al., 1997; Heniquez et al., 2003; Bour et al., 2007). The substrates of AOC2 include 2-phenylethylamine, tryptamine and p-tyramine, but it does not oxidize histamine (Finney et al., 2014). *AOC3* is highly expressed in adipocytes, smooth muscle cells, and endothelial cells, and is abundantly present in the lungs, aorta, liver, and ileum (Kurkijärvi et al., 1998).

While the roles of CuAOs are slowly being clarified, studies on these enzymes remain confined to a limited number of model organisms, including humans and *A. thaliana*. Currently, our understanding of the origin and evolution of CuAOs is sparse, and a comprehensive investigation into CuAOs across both plant and animal kingdoms is still missing. In this study, we explored the evolutionary path of CuAOs by examining the complete genomic sequences from various plants and animals. As far as we are aware, this represents the most thorough phylogenetic analysis of CuAOs conducted so far. This research offers an in-depth view of the evolution of CuAOs, examining their origins, evolutionary processes, and functional diversity, while also establishing a strong basis for future studies on functional resolution and molecular evolution.

## Results

### Identification and distribution of *CuAO* genes

In order to comprehensively identify CuAO in plants and animals, we selected a diverse range of 188 plant species and 79 animal species, including rhodophytes, chlorophytes, charophytes, bryophytes (hornworts, mosses, liverworts), ferns, lycophytes, gymnosperms, basal angiosperms, angiosperms, invertebrates and vertebrates (**Table 1**, **Supplementary Table S1**). Through homology search and domain prediction, a total of 1214 candidate protein sequences of CuAO were retrieved, including 950 plant sequences and 264 animal sequences (**Table 1**, **Supplementary Table S2**). CuAO copy numbers are conserved among different animal lineages, while they vary significantly among different plant lineages (**Table 1**). The average count of CuAOs was 2 in rhodophytes, 1.6 in chlorophytes, 1.8 in charophytes, 1.29 in hornworts, 3.38 in mosses, 2.59 in liverworts, 3 in ferns, 3.14 in lycophytes, 4.07 in gymnosperms, 2 in basal angiosperms, 2.36 in magnoliids, 5.57 in monocots, and 7.53 in eudicots. The copy numbers of CuAO were lower in algal species, higher in land plants, and significantly elevated in seed plants. This change may be closely related to the gradual increase in biological complexity or the whole genome duplication (WGD) events experienced during plant evolution. Interestingly, most animal lineages have a relatively stable number of CuAOs, typically ranging from 3 to 4.

**Table 1:** The number of *CuAO* in plant species and animal species.

Taxonomy	Number of species	Number of *CuAO*	Average number of *CuAO* per species
Rhodophytes	2	4	2.00
Chlorophytes	5	8	1.60
Charophytes	5	9	1.80
hornworts	7	9	1.29
mosses	8	27	3.38
liverworts	17	44	2.59
Lycophytes	7	22	3.14
Ferns	1	3	3.00
Gymnosperms	15	61	4.07
Basal angiosperms	3	6	2.00
Magnoliids	14	33	2.36
Monocots	30	167	5.57
Eudicots	74	557	7.53
Brassicaceae	36	273	7.58
invertebrates	15	53	3.53
vertebrates	64	211	3.30

### Evolutionary origin of CuAOs in plants and animals

**Figure 1:**
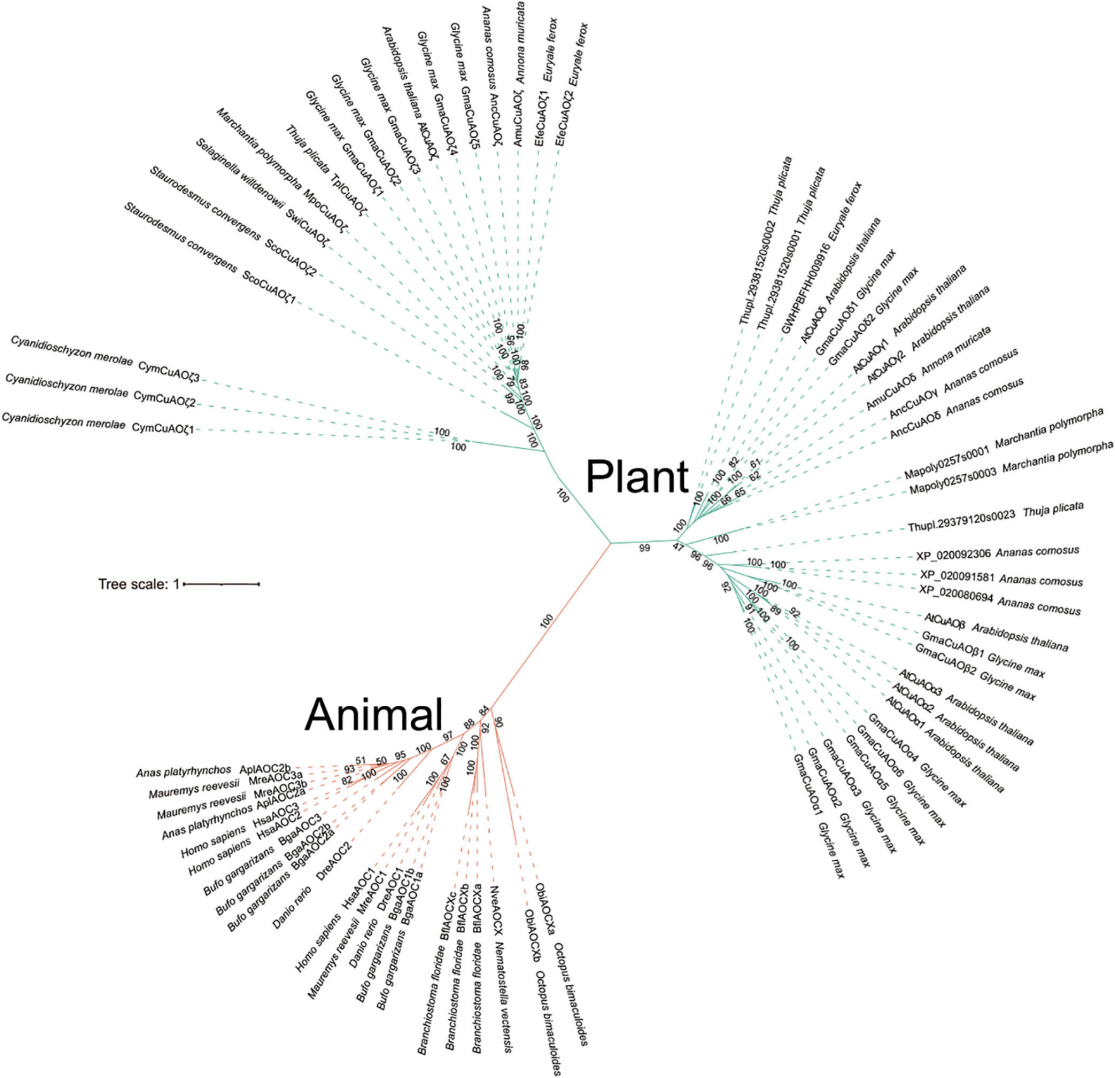
Phylogenetic analysis of the *CuAO* family members in representative plant and animal genomes. The ML tree was built using the full-length protein sequences by IQ-TREE2 with the MFP model. Scale bars indicate substitutions per site. Distinct branch colors represent various plant and animal lineages. The topological structure of the phylogenetic tree clearly divides the *CuAOs* from plants and animals into two distinct clades. The *CuAOs* in plants have undergone significant expansion, forming two clades.

To further explore the evolutionary origin of CuAOs, we selected representative species and constructed phylogenetic trees with IQ-TREE2 tool. The phylogenetic tree topologies distinctly categorized CuAOs from plants and animals into two separate clades, suggesting that the origin of CuAOs predates the divergence between plants and animals (**Figure 1**). Additionally, the phylogenetic tree shows that CuAOs underwent a distinct expansion in plants, forming two branches.

### Phylogenetic classification of plant CuAOs

**Figure 2:**
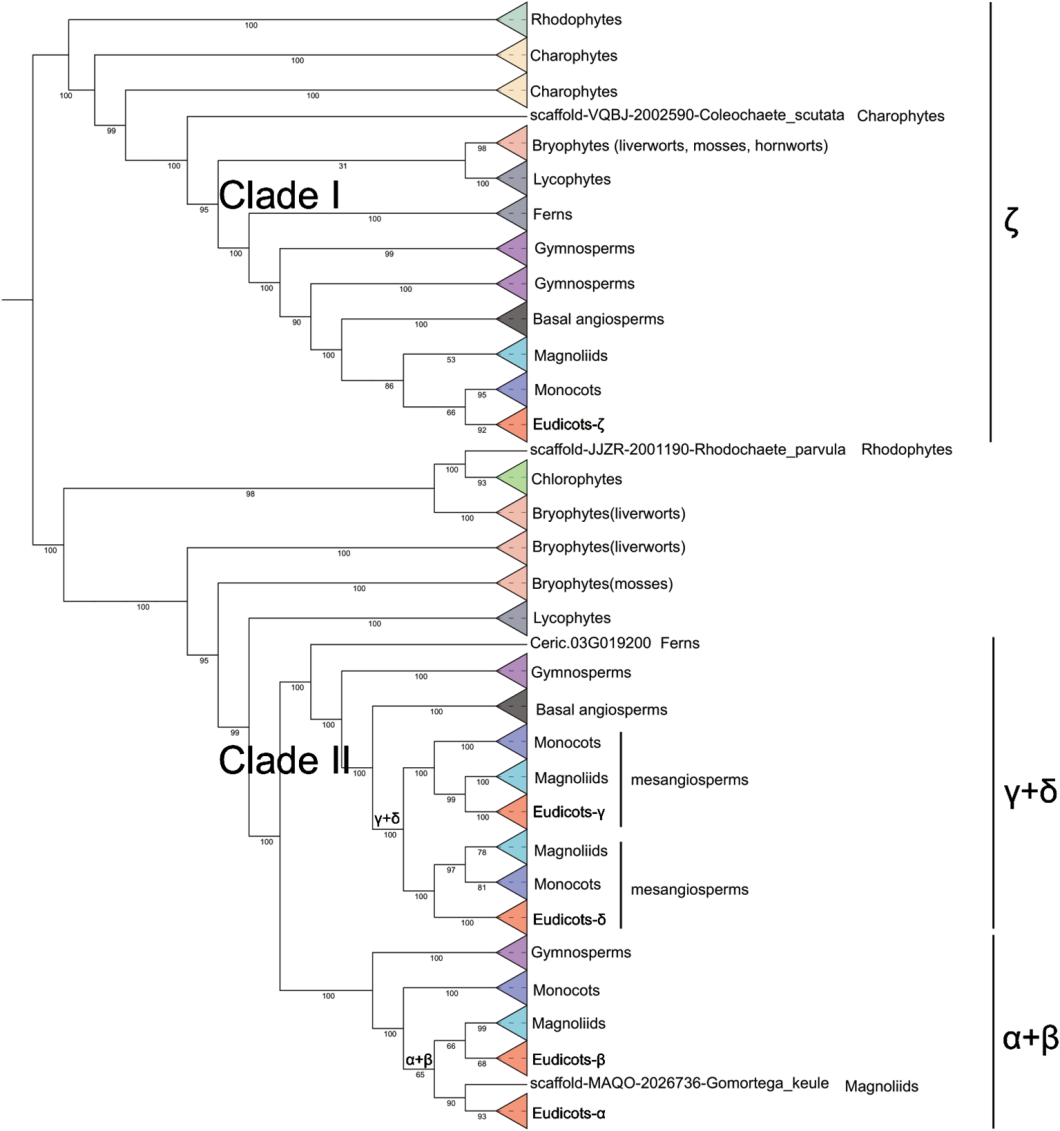
Phylogenetic relationship of *CuAO* genes in plants. The ML tree was built using the full-length protein sequences by IQ-TREE2 with the MFP model. The phylogenetic tree is simplified using the iTOL tool. Different colored triangles represent different plant lineages. *CuAO* expanded and split into two clades in the most recent common ancestor of plants: clade I and clade II. Clade I, also known as the ζ clade, is a monophyletic clade. Clade II expanded after the differentiation of vascular plants into lycophytes and ferns, forming the γ + δ and α + β sub-clades.

An IQ tree was constructed for CuAO proteins from plant lineages including rhodophytes, chlorophytes, charophytes, bryophytes (hornworts, mosses, liverworts), ferns, lycophytes, gymnosperms, basal angiosperms, angiosperms (**Figure 2**, **Supplementary Figure S1**). The phylogenetic tree shows that CuAO expanded and split into two branches in the most recent common ancestor of plants: clade I and clade II. Clade I is also known as the ζ branch and is a monophyletic branch, indicating that its members share a common ancestor. They evolved independently during radiation of plants. Clade II expanded after the division of vascular plants into the lycophytes and ferns, forming the subbranches of γ + δ and α + β. The differentiation of γ and δ occurred in the common ancestor of the mesangiosperms, while the differentiation of α and β occurred in the common ancestor of the eudicots and magnoliids. Clade I lost the green algae, and Clade II lost hornwort.

### Phylogenetic classification of the animal CuAOs

**Figure 3:**
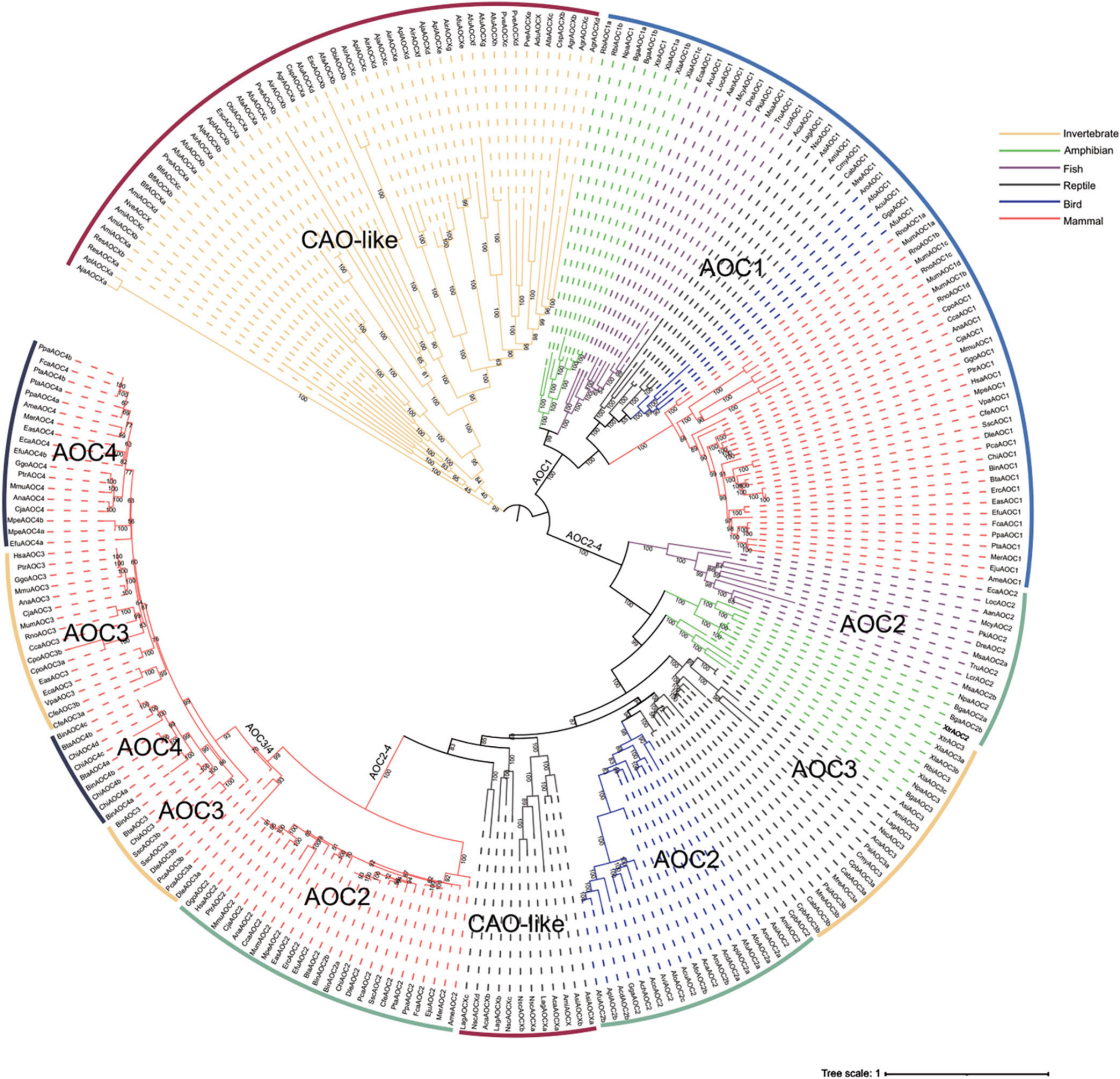
Phylogenetic relationship of *CuAOs* in animals. The phylogenetic tree was built using IQ-TREE2. Distinct branch colors represent various animal lineages. Among invertebrates, *CuAOs* of the Coelenterata, Mollusca and Leptocardii form a highly supported monophyletic clade. In vertebrates, *CuAOs* can be divided into two major clades, namely *AOC1* and *AOC2-4*. Within *AOC1*, mammals and non-mammals form a monophyletic clade, while in *AOC2-4*, mammals and non-mammals are separated.

To better understand the evolutionary relationships of animal CuAOs, we constructed an IQ tree using the CuAO sequences of 79 animals (15 invertebrates and 64 vertebrates) (**Figure 3**). In invertebrates, the CuAO from Coelenterata, Mollusca, and Leptocardii formed highly supported monophyletic clades. Vertebrates include mammals, birds, reptiles, amphibians and fish. The vertebrate CuAO can be divided into two main branches, AOC1 and AOC2-4. In AOC1, mammals and non-mammals form a monophyletic branch, while in AOC2-4, mammals and non-mammals are separated. Non-mammalian AOC2 and AOC3 form their own subbranches, while mammalian AOC2-4 further divide into the subbranches of AOC2 and AOC3/4. Interestingly, the differentiation of AOC3 and AOC4 in mammals occurs in species-specific ways, which may be due to different evolutionary pressures. AOC1 and AOC2 are present in all vertebrates, AOC3 is present in amphibians, reptiles, and mammals, and AOC4 is unique to mammals. CAO-like is present in invertebrates and reptiles.

### Phylogenetic classification of CuAOs in Brassicaceae

**Figure 4:**
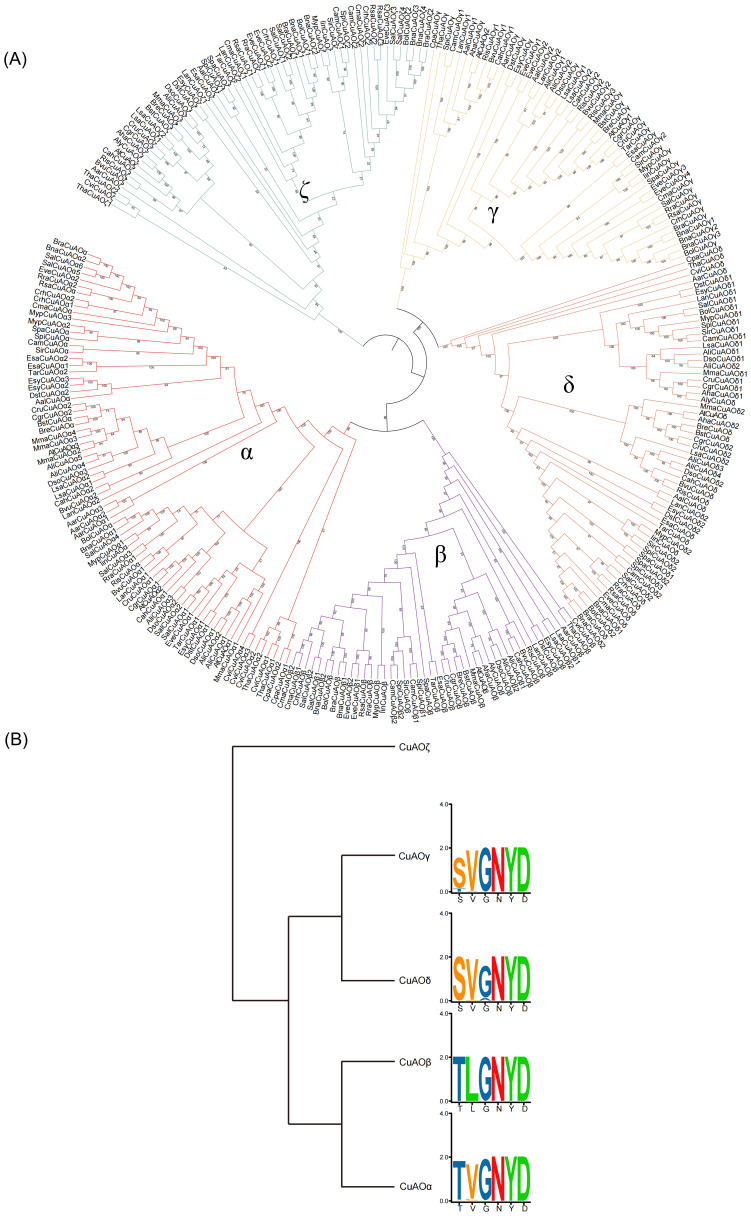
Phylogenetic classification of *CuAOs* in Brassicaceae. **(A)** The phylogenetic tree was built using IQ-TREE2. Distinct branch colors represent various plant lineages. All five subclades of Brassicaceae are monophyletic. **(B)** The Brassicaceae *CuAOζ* clade completely lacks the T/S-X_1_-X_2_-N-Y-D active site motif. The specific preference of residue X_1_ corresponds to the phylogenetic classification of Brassicaceae *CuAOα* and *CuAOβ*.

Certain species within the Brassicaceae family are widely cultivated and possess considerable economic importance. To further elucidate the systematic relationships within the Brassicaceae CuAO family, we constructed a phylogenetic tree using 36 Brassicaceae plants and three sister groups (*Carica papaya*, *Tarenaya hassleriana*, *Cleome violacea*) with a total of 291 CuAOs (**Figure 4**). The five subbranches of the Brassicaceae are all monophyletic branches, and the α branch has a significantly higher number of CuAOs than the other subbranches (**Figure 4A**).

Previous studies have shown that the variable residues X_1_ and X_2_ in the T/S-X_1_-X_2_-N-Y-D active site motif of proteases follow the substrate preference of mammalian CuAOs, which helps distinguish their subfamilies (Salminen et al., 1998; Kivi et al., 2009; Lopes de Carvalho et al., 2019). This leads us to believe that the residues at positions X_1_ and X_2_ may help distinguishing between the Brassicaceae CuAO subfamilies. The results show that the Brassicaceae CuAOζ branch lacks the T/S-X_1_-X_2_-N-Y-D active site motif in its entirety, which may affect the function of the enzyme or its role in metabolic pathways, suggesting that this branch has different biological functions from other branches with active sites (**Figure 4B**). CuAOγ and CuAOδ tend to have the first amino acid in the active site motif more biased towards serine (S), while CuAOβ and CuAOα are more biased towards threonine (T). The active site motifs of CuAOγ and CuAOδ are highly conserved, while those of CuAOβ and CuAOα differ. In CuAOβ, the residue at position X_1_ is always leucine (L), while in CuAOα, the residue is more inclined towards valine (V). Therefore, the specific bias of residue X_1_ corresponds with the phylogenetic classification of the Brassicaceae CuAOα and CuAOβ.

### Expansion of CuAOs during plant and animal evolution

**Figure 5:**
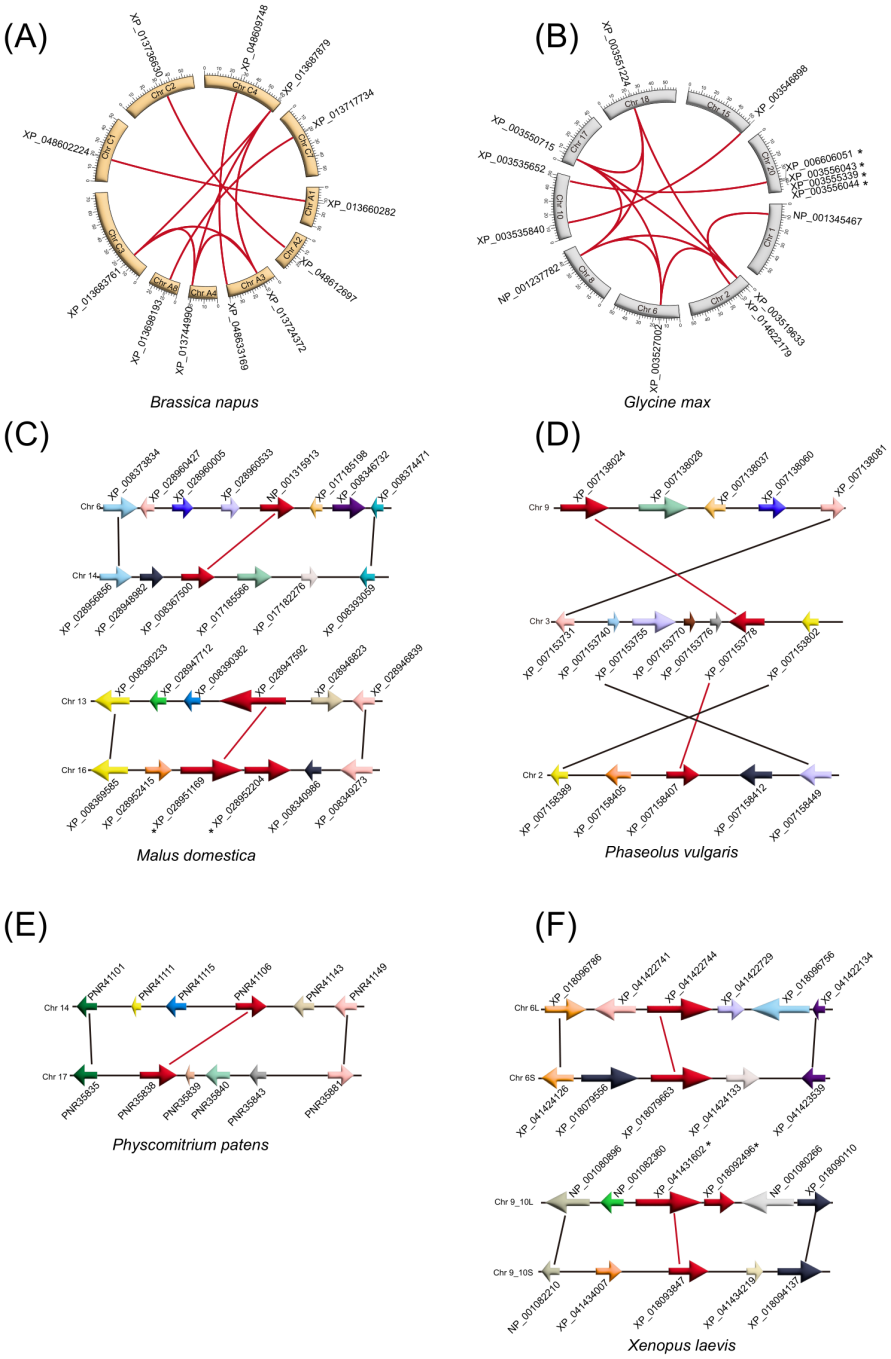
Intraspecies syntenic relationships of *CuAO* genes in representative plants and animals. The syntenic paralog of *CuAO* genes are connected by red lines. The asterisk indicates tandem duplicate pairs. The expansion of *CuAO* genes in different plant species is driven by multiple mechanisms acting in concert. In animal species, the vast majority are only tandem duplications. **(A)** *Brassica napus*; **(B)** *Glycine max*; **(C)** *Malus domestica*; **(D)** *Phaseolus vulgaris*; **(E)** *Physcomitrium patens*; **(F)** *Xenopus laevis*.

In most species, tandem and segmental duplications serve as essential forces driving evolution (Peng et al., 2021). To explore the expansion of *CuAO* genes in plants and animals, we conducted a synteny analysis (**Figure 5**, **Supplementary Figure S2**, **Supplementary Table S3**). 10, 12, 2, 4, 3, 2, 1, 16, 7, and 2 pairs of segmental duplication genes were identified in *B. napus*, *G. max*, *M. domestica*, *M. acuminata*, *P. somniferum*, *P. vulgaris*, *P. patens*, *T. aestivum*, *P. virgatum*, and *X. laevis*, respectively. Furthermore, tandem duplication resulted in 4, 3, 2, 2, 4, 2, 2, 2, 5, 2, 6, 2, 2, 6, 4, 5, 6, 3, 2, 6, 2, 5, 2, 2, and 2 additional *CuAO* genes in *A. comosus*, *A. thaliana*, *C. papaya*, *G. biloba*, *G. max*, *M. domestica*, *O. sativa*, *P. somniferum*, *P. vulgaris*, *S. tuberosum*, *T. cacao*, *V. vinifera*, *P. virgatum*, *B. indicus*, *B. taurus*, *B. gargarizans*, *C. hircus*, *G. gorilla*, *H. sapiens*, *M. musculus*, *N. parkeri*, *N. scutatus*, *R. bivittatum*, *X. laevis*, and *X. tropicalis*, respectively. Various mechanisms contribute to the expansion of *CuAO* genes in different plant species. Segmental duplication primarily drives this expansion in *G. max*, *P. somniferum*, and *P. virgatum*, while tandem duplication is the main mechanism for *P. vulgaris*. Segmental duplication occurs only in *B. napus*, *M. acuminata*, *P. patens*, and *T. aestivum*, whereas tandem duplication is exclusive to *A. thaliana*, *A. comosus*, *C. papaya*, *G. biloba*, *O. sativa*, *S. tuberosum*, *T. cacao*, and *V. vinifera*. Interestingly, in animal species, the vast majority only have tandem duplication. This emphasizes that different plant species adopt diverse evolutionary strategies to expand the *CuAO* genes, while animal species tend to have tandem duplication.

The analysis of Ka/Ks ratios (the rate of non-synonymous to synonymous substitutions) was performed to evaluate selection pressure on gene evolution (Zhang et al., 2024). A Ka/Ks ratio above 1 indicates positive selection, while a ratio below 1 suggests purifying or negative selection (Zhang et al., 2024). These results revealed that the Ka/Ks ratios for all CuAO paralogs were below 1, suggesting that they underwent purifying selection throughout their evolution (**Supplementary Table S4**).

**Figure 6:**
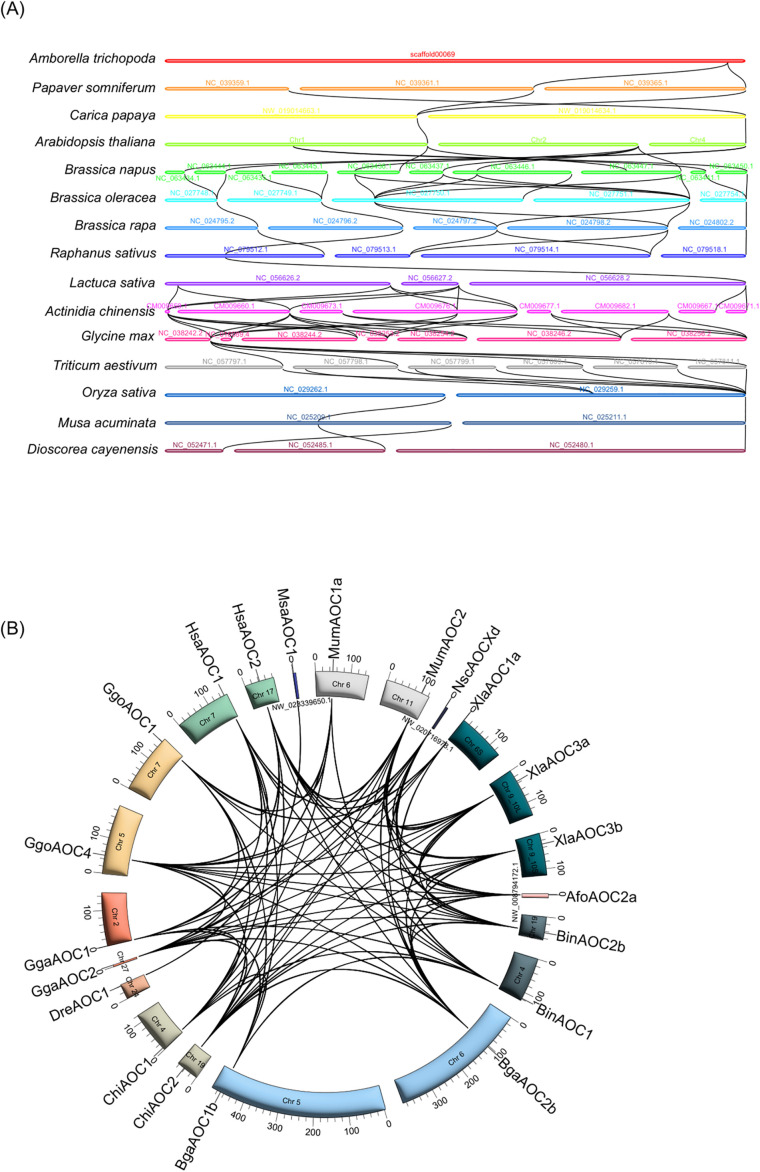
Interspecies syntenic relationships of *CuAO* genes in plants and animals. The syntenic paralog of *CuAO* genes are connected by black lines. **(A)** Plant *CuAOs* exhibit one-to-one or one-to-many homology. **(B)** In vertebrates, *AOC1* only shows interspecies orthology with *AOC1*, while *AOC2*, *AOC3*, *AOC4* and *AOCX* display interspecies orthology among themselves.

To delve deeper into the evolutionary relationships of the *CuAO* gene, inter-species synteny analyses were conducted on 15 plant genomes and 12 animal genomes (**Figure 6**, **Supplementary Table S5**). The CuAO homologous genes of individual plants demonstrated one-to-one collinear relationships between *C. papaya* and *A. thaliana*, *R. sativus* and *L. sativa*, *O. sativa* and *M. acuminata*, *M. acuminata* and *D. cayenensis* (**Figure 6A**). In addition, either one-to-many or many-to-one homozygosity was identified between *A. trichopoda* and *P. somniferum*, *P. somniferum* and *C. papaya*, *A. thaliana* and *B. napus*, *B. napus* and *B. oleracea*, *B. oleracea* and *B. rapa*, *B. rapa* and *R. sativus*, *L. sativa* and *A. chinensis*, *A. chinensis* and *G. max*, *G. max* and *T. aestivum*, *T. aestivum* and *O. sativa* (**Figure 6A**). Interestingly, in vertebrates, AOC1 shows interspecies synteny only with AOC1, while AOC2, AOC3, AOC4, and AOCX show interspecies synteny among themselves (**Figure 6B**).

### Expression patterns of *CuAOs* in key evolutionary lineages of plants and animals

**Figure 7:**
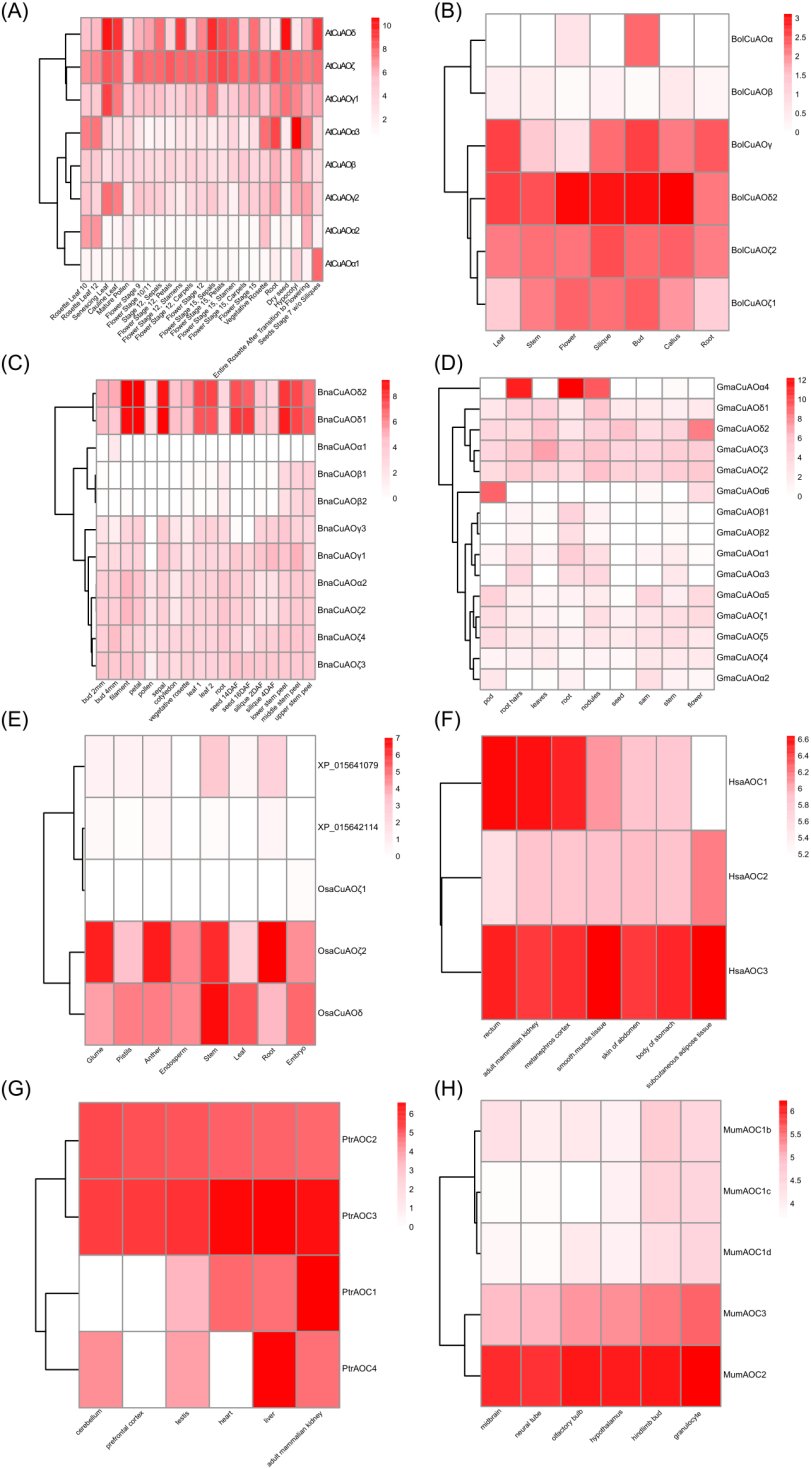
Expression of *CuAOs* in plants and animals. **(A)**, *A. thaliana*; **(B)**, *B. oleracea*; **(C)**, *B. napus*; **(D)**, *G. max*; **(E)**, *O. sativa*; **(F)**, *H. sapiens*; **(G)**, *P. troglodytes*; **(H)**, *M. musculus*. The expression of duplicated *CuAO* genes in Brassicaceae varies in different tissues. In animals, *AOC2* and *AOC3* are widely expressed in most tissues.

To investigate the expression of *CuAO* genes, we performed a gene expression analysis of *CuAOs* in eight representative species from plants and animals: *A. thaliana*, *B. napus*, *B. oleracea*, *G. max*, *O. sativa*, *H. sapiens*, *M. musculus*, and *P. troglodytes* (**Figure 7**). In *A. thaliana*, we examined 23 different tissues and developmental stages (**Figure 7A**). *AtCuAOδ*, *AtCuAOζ*, *AtCuAOγ1*, *AtCuAOα3*, *AtCuAOβ*, and *AtCuAOγ2* are widely expressed in various tissues, with *AtCuAOζ* showing significantly higher expression than the other *CuAO* genes. Interestingly, in various tissues, *AtCuAOγ1* is generally expressed at higher levels than *AtCuAOγ2*, while *AtCuAOα3* shows significantly higher expression than both *AtCuAOα2* and *AtCuAOα1*. Notably, *AtCuAOα1* is highly expressed specifically in siliques, whereas *AtCuAOα2* is predominantly expressed in rosette leaves. In *B. oleracea*, *BolCuAOδ2*, *BolCuAOζ2*, and *BolCuAOζ1* are widely expressed, with *BolCuAOδ2* showing higher expression levels (**Figure 7B**). In leaves, siliques, and callus, the expression of *BolCuAOζ2* exceeds that of *BolCuAOζ1*, while in flowers and buds, *BolCuAOζ1* is more highly expressed than *BolCuAOζ2*. Additionally, *BolCuAOα* is specifically highly expressed in buds. *B. napus* and *G. max* have higher number of *CuAOs*, but their expression levels are generally low across all detected tissues (**Figures 7C, D**). *BnaCuAOδ1* and *BnaCuAOδ2* exhibit highly similar expression patterns, showing significantly higher specific expression than other genes in filaments, petals, sepals, leaves, seeds, and stem peels (**Figure 7C**). *GmaCuAOα4* is specifically expressed at high levels in root hairs, roots, and nodules, while *GmaCuAOα6* is specifically expressed at high levels in pods (**Figure 7D**). In rice, *OsaCuAOζ2* is specifically highly expressed in the glumes, anthers, stems, and roots, while *OsaCuAOδ* is specifically highly expressed in the stems (**Figure 7E**). In summary, duplicated *CuAO* genes in Brassicaceae exhibit differing expression across various tissues, indicating functional divergence among these paralogs. In soybean and rice, their specific expression suggests roles in different growth stages or plant tissues, potentially involving development, growth, and nutrient metabolism.

Most animal lineages have a relatively few *CuAOs*, usually 3 to 4. *AOC2* and *AOC3* are widely expressed in most tissues (**Figures 7F-H**). In humans and chimpanzees, *AOC3* is expressed significantly more than *AOC2* (**Figures 7F, G**), while in mice, *MumAOC2* is expressed significantly more than *MumAOC3* (**Figure 7H**). The expression of *HsaAOC1* is tissue-specific, with high specific expression in the rectum, adult mammalian kidney, and metanephros cortex in humans and in the adult mammalian kidney in chimpanzees (**Figure 7F-G**). Additionally, *PtrAOC4* is specifically highly expressed in the liver of chimpanzees.

## Discussion

**Figure 8:**
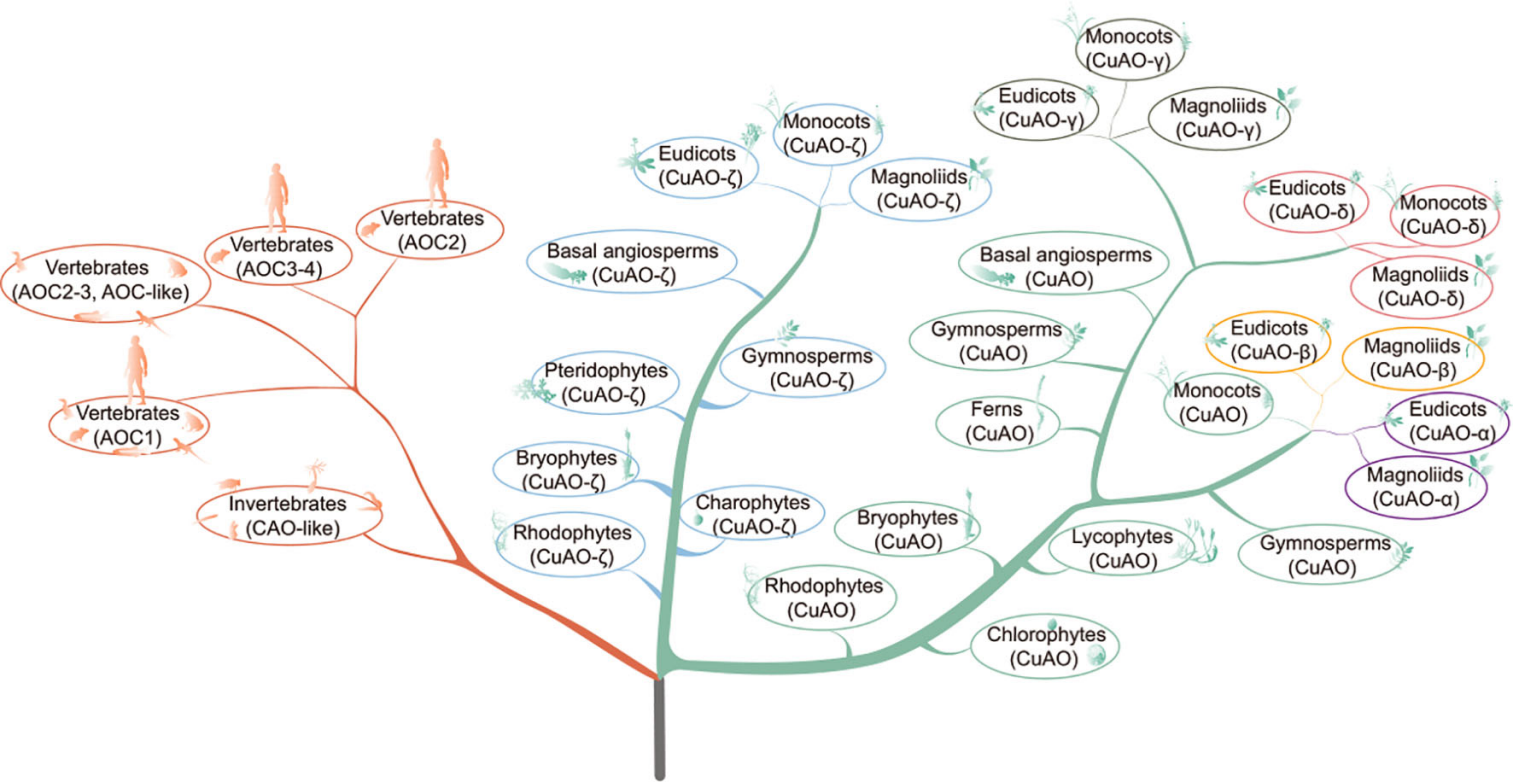
A proposed evolutionary model of *CuAOs* in animals and plants. The model is based on the phylogeny of *CuAOs* and the cladogram of animals and plants. *CuAOs* from plants and animals are clearly divided into two distinct clades. In plants, *CuAOs* have undergone significant expansion and further evolved into two independent clades.

This study conducted phylogenetic classification and examination of the characteristics of thousands of *CuAOs* in hundreds of plant and animal species, deepening our understanding of *CuAOs* in plants and animals, particularly those in angiosperms and vertebrates. The phylogenetic perspective aids in uncovering the molecular and biological roles of different CuAO proteins (**Figure 8**).

### Phylogenetic relationship of *CuAOs* in plant and animal

Since genome-based identification of CuAO proteins might overlook certain proteins due to the quality of genome assembly and annotation accuracy, we incorporated numerous species within each phylogenetic group to mitigate the effects of any potentially missing proteins. Our search for the CuAO proteins revealed that the *CuAO* gene family is widely distributed across both plant and animal lineages, with varying copy numbers in different plant species and a more conservative distribution in animal species, with approximately 3-4 copies per species (**Table 1**). Our study suggests that the origin of *CuAOs* predates the divergence of plants and animals, implying that the bioammonia degradation pathway was established in the common ancestor of plants and animals (**Figure 1**) (Brazeau et al., 2004; Murakawa et al., 2019). After the divergence of plant and animal species, *CuAOs* underwent significant expansion in plants, forming two branches (Clade I and Clade II) (**Figure 2**). It is worth noting that, consistent with previous research results, we identified a *CuAO* gene located in the ζ branch in *Spirodela polyrhiza* (Upadhyay et al., 2024b). Interestingly, in the ζ branch, most monocots have multiple copies of the *CuAO* gene, while a few monocots (*S. polyrhiza*, *A. americanus*, *K. littledalei*, *Z. mays*, *B. distachyon*) contain only a single copy (**Supplementary Figure S1**). Previous studies have shown that mammalian CuAOs can be classified based on the two residues X_1_ and X_2_ in the active site motif T/S-X_1_-X_2_-N-Y-D (Lopes de Carvalho et al., 2019). Our study shows that Clade I is a monophyletic branch that has lost the T/S-X_1_-X_2_-N-Y-D active site motif, which may affect the function of the enzyme or its role in the metabolic pathway, suggesting that Clade I is functionally diverse from Clade II (**Figures 2**, **4**). In previous studies, the plant *CuAOs* were divided into three subfamilies (clades I-III), clade I consists of the α and β subgroups, clade II consists of the γ and δ subgroups, and clade III consists of the ζ subgroup (Tavladoraki et al., 2016). In our study, Clade II further expanded during evolution and formed four major branches: α, β, γ, and δ. CuAOγ and CuAOδ tend to have serine (S) as the first amino acid in the active site motif, while CuAOβ and CuAOα are more inclined towards threonine (T) (**Figure 4B**). The active site motifs of CuAOγ and CuAOδ are highly conserved, but those of CuAOβ and CuAOα differ. In CuAOβ, the residue at position X_1_ is always leucine (L), while in CuAOα, it is more likely to be valine (V). Thus, the specific bias of residue X_1_ correlates with the phylogenetic classification of CuAOα and CuAOβ (**Figure 4**).

In the phylogenetic analysis of vertebrate *CuAOs*, both mammalian and non-mammalian AOC1 proteins are grouped in the same clade, showing a robust bootstrap support of 100% (**Figure 3**). In contrast to AOC1s, non-mammalian AOC2-4 proteins deviate from their mammalian equivalents with a bootstrap value of 100% as well (**Figure 3**). Non-mammalian AOC2 and AOC3 proteins form distinct sub-branches, while mammalian AOC2-4 proteins are further classified into separate sub-branches for AOC2 and a combined group for AOC3/AOC4, both presenting high bootstrap values. These results are in line with previous studies (Lopes de Carvalho et al., 2019). Interestingly, the differentiation between mammalian AOC3 and AOC4 proteins occurs in a species-specific manner, perhaps due to different evolutionary pressures. Furthermore, unlike previous studies, our research showed that cartilaginous fish have not only AOC1 but also AOC2 (Lopes de Carvalho et al., 2019).

### Insights into *CuAO* evolution and functional diversification

To understand the evolutionary implications of the phylogenetic architecture of the *CuAO* family, it is optimal to cross-reference the phylogenetic relationships of *CuAOs* and their functions. Repeated events frequently cause the expansion and functional diversification of gene families (Peng et al., 2024). Generally, *CuAOs* within the same clade have similar and specific functions. *CuAOδ* is highly expressed in flowers, and this is the case in *A. thaliana*, *B. oleracea*, *B. napus*, *G. max* and *O. sativa* (**Figure 7A-E**). Studies have shown that *Malus domestica CuAOδ* (*MdAO2*) contributes to the formation of flower fragance (Zarei et al., 2015). Furthermore, different members of the same clade might have functional specialization or redundancy. For example, the expression of *AtCuAOα3* is significantly higher than that of *AtCuAOα1* and *AtCuAOα2*, and the expression of *AtCuAOγ1* is much higher than that of *AtCuAOγ2*; while the expression patterns of *BolCuAOζ1* and *BolCuAOζ2* are alike, and so are those of *BnaCuAOδ1* and *BnaCuAOδ2* (**Figure 7A-E**). *SlCuAO1* (*SlyCuAOβ3*), *SlCuAO2* (*SlyCuAOζ1*), *SlCuAO3* (*SlyCuAOζ2*), *SlCuAO4* (*SlyCuAOζ3*), *SlCuAO6* (*SlyCuAOβ2*) and *SlCuAO7* (*SlyCuAOβ1*) in *S. lycopersicum* are mainly specifically expressed in roots, while *SlCuAO5* (*SlyCuAOδ*) is specifically expressed in fruits, and *SlCuAO8* (*SlyCuAOα*) is mainly expressed in flowers (Upadhyay et al., 2024a). The *CuAO* gene family in tomato shows obvious tissue-specific expression: the β and ζ subtypes are mainly expressed in roots, the δ subtype is enriched in fruits, and the α subtype is highly expressed in flowers. This branch-specific expression pattern reflects the functional differentiation of the CuAO gene family.

Compared with *A. thaliana CuAO*, human, mouse, and chimpanzee *CuAO* isoforms show significant functional differences. For example, in humans and chimpanzees, the expression level of *AOC3* is significantly higher than that of *AOC2* and *AOC1*, while in mice, the expression level of *AOC2* is significantly higher than that of *AOC3* and *AOC1* (**Figure 7F-H**). The expression of *AOC1* is tissue-specific and is highly expressed in the kidneys (Schwelberger, 2006). *AOC1* gene encodes diamine oxidase (Elmore et al., 2002), and human *AOC1* is thought to have *in vivo* functions in cell proliferation, inflammation, allergic reactions, and ischemia (McGrath et al., 2009). *AOC2* gene encodes retinal-specific amine oxidase, and human *AOC2* is thought to play a role in hereditary retinal diseases (Kaitaniemi et al., 2009). *AOC3* gene encodes vascular adhesion protein-1, and human *AOC3* expression is upregulated during inflammation and plays an important role in the migration of white blood cells from the blood into tissues (Salmi et al., 1993; Smith et al., 1998; Jalkanen et al., 2007). These difference suggest different functions of these three isoforms.

## Materials and methods

### Data sources and sequence acquisition

We selected 267 species and obtained their genome and proteome sequences from public databases like NCBI (http://www.ncbi.nlm.nih.gov/), Ensembl Plants (http://plants.ensembl.org/index.html), and BRAD (http://brassicadb.cn/#/) (**Supplementary Table S1**). To identify *CuAO* genes in animals and plants, we first performed an HMMER search (E-value = 1e-10) using the Hidden Markov Model profile of the CuAO domain (PF01179) in local databases (Potter et al., 2018; Chen et al., 2022). The HMMER parameters are set as follows: hmmsearch -E 1e-10 PF01179.hmm protein.fa. We also used BLASTP with *A. thaliana* CuAO amino acid sequences against the protein database, setting an E-value threshold of less than 1e-6. The putative CuAOs were further validated using online tools CDD, HMM, and SMART (Marchler-Bauer et al., 2015; Letunic et al., 2021).

### Multiple sequence alignment (MSA) and phylogenetic analysis

Multiple sequence alignments of CuAO were performed using Muscle5 (Edgar, 2022). Phylogenetic trees were generated from the full-length protein sequences of CuAO. The maximum likelihood (ML) tree was constructed with IQ-TREE2 using parameters ‘-m MFP -B 1000’ and 1000 bootstrap replicates (Minh et al., 2020). The consensus tree topology was visualized using iTOL (Letunic and Bork, 2024).

### Synteny analysis

To identify homologous pairs in a specific species, we used the all-to-all BLASTP method. Syntenic blocks were inferred using MCScanX with default parameters, including an E-value threshold of 1e-10 and a minimum of 5 BLAST hits (Wang et al., 2012). The resulting gene arrangement was visualized with CIRCOS, linking potential duplicated genes to show their connections (Krzywinski et al., 2009). The duplicated *CuAO* gene pairs were subjected to TBtools software for calculation of synonymous (Ka) and non-synonymous (Ks) substitution rates (Chen et al., 2023).

### Expression analysis of CuAO genes

Expression data for ten species were downloaded from public databases, including *A. thaliana* (The Arabidopsis Information Resource), *B. napus* (https://yanglab.hzau.edu.cn/BnIR), *B. oleracea* (http://bogdb.com/), *G. max* (https://www.soybase.org/), *O. sativa* (http://rice.uga.edu/index.shtml), *H. sapiens* (https://www.bgee.org/), *M. musculus* (https://www.bgee.org/), and *P. troglodytes* (https://www.bgee.org/) (**Supplementary Table S6**). Heat map was established using the R package “pheatmap” (https://github.com/raivokolde/pheatmap).

## Data Availability

The original contributions presented in the study are included in the article/**Supplementary Material**. Further inquiries can be directed to the corresponding author/s.

## Supplementary material

Supplementary Table 1 List of complete predicted proteomes databases used in this study.

Supplementary Table 2 The identified CuAO protein.

Supplementary Table 3 Segmental duplication and tandem gene.

Supplementary Table 4 The Ka/Ks analysis of *CuAO* genes.

Supplementary Table 5 Segmental duplication gene pairs of CuAOs in interspecies.

Supplementary Table 6 Source of expression data.

Supplementary Figure 1 Phylogenetic relationship of representative *CuAO* genes from plants. The phylogenetic tree was built using IQ-TREE2. Distinct branch colors represent various plant lineages.

Supplementary Figure 2 Intraspecies syntenic relationships of *CuAO* genes in representative plants. The syntenic paralogs of *CuAO* genes are connected by black lines. The asterisk indicates tandem duplicate pairs.

## References

[B1] Boomsma F. Bhaggoe U. M. van der Houwen A. M. van den Meiracker A. H. (2003). Plasma semicarbazide-sensitive amine oxidase in human (patho)physiology. Biochim. Biophys. Acta 1647, 48–54. doi: 10.1016/S1570-9639(03)00047-5 12686107

[B2] Bour S. Daviaud D. Gres S. Lefort C. Prévot D. Zorzano A.. (2007). Adipogenesis-related increase of semicarbazide-sensitive amine oxidase and monoamine oxidase in human adipocytes. Biochimie 89, 916–925. doi: 10.1016/j.biochi.2007.02.013 17400359

[B3] Brazeau B. J. Johnson B. J. Wilmot C. M. (2004). Copper-containing amine oxidases. Biogenesis and catalysis; a structural perspective. Arch. Biochem. Biophys. 428, 22–31. doi: 10.1016/j.abb.2004.03.034 15234266

[B4] Chen H. Wang T. He X. Cai X. Lin R. Liang J.. (2022). BRAD V3.0: an upgraded Brassicaceae database. Nucleic Acids Res. 50, D1432–d1441. doi: 10.1093/nar/gkab1057 34755871 PMC8728314

[B5] Chen C. Wu Y. Li J. Wang X. Zeng Z. Xu J.. (2023). TBtools-II: A “one for all, all for one” bioinformatics platform for biological big-data mining. Mol. Plant 16, 1733–1742. doi: 10.1016/j.molp.2023.09.010 37740491

[B6] Dunkel P. Gelain A. Barlocco D. Haider N. Gyires K. Sperlágh B.. (2008). Semicarbazide-sensitive amine oxidase/vascular adhesion protein 1: recent developments concerning substrates and inhibitors of a promising therapeutic target. Curr. Med. Chem. 15, 1827–1839. doi: 10.2174/092986708785133022 18691041

[B7] Edgar R. C. (2022). Muscle5: High-accuracy alignment ensembles enable unbiased assessments of sequence homology and phylogeny. Nat. Commun. 13, 6968. doi: 10.1038/s41467-022-34630-w 36379955 PMC9664440

[B8] Elmore B. O. Bollinger J. A. Dooley D. M. (2002). Human kidney diamine oxidase: heterologous expression, purification, and characterization. J. Biol. Inorganic. Chem. 7, 565–579. doi: 10.1007/s00775-001-0331-1 12072962

[B9] Finney J. Moon H. J. Ronnebaum T. Lantz M. Mure M. (2014). Human copper-dependent amine oxidases. Arch. Biochem. Biophys. 546, 19–32. doi: 10.1016/j.abb.2013.12.022 24407025 PMC5995473

[B10] Fortes A. M. Agudelo-Romero P. Pimentel D. Alkan N. (2019). Transcriptional modulation of polyamine metabolism in fruit species under abiotic and biotic stress. Front. Plant Sci. 10, 816. doi: 10.3389/fpls.2019.00816 31333688 PMC6614878

[B11] Fraudentali I. Ghuge S. A. Carucci A. Tavladoraki P. Angelini R. Cona A.. (2019). The copper amine oxidase atCuAOδ Participates in abscisic acid-induced stomatal closure in arabidopsis. Plants (Basel Switzerland). 8, 1–15. doi: 10.3390/plants8060183 PMC6630932 31226798

[B12] Fraudentali I. Ghuge S. A. Carucci A. Tavladoraki P. Angelini R. Rodrigues-Pousada R. A.. (2020a). Developmental, hormone- and stress-modulated expression profiles of four members of the Arabidopsis copper-amine oxidase gene family. Plant Physiol. Biochem.: PPB 147, 141–160. doi: 10.1016/j.plaphy.2019.11.037 31862580

[B13] Fraudentali I. Pedalino C. D’Incà R. Tavladoraki P. Angelini R. Cona A. (2023). Distinct role of AtCuAOβ- and RBOHD-driven H(2)O(2) production in wound-induced local and systemic leaf-to-leaf and root-to-leaf stomatal closure. Front. Plant Sci. 14, 1154431. doi: 10.3389/fpls.2023.1154431 37152169 PMC10160378

[B14] Fraudentali I. Rodrigues-Pousada R. A. Angelini R. Ghuge S. A. Cona A. (2021). Plant copper amine oxidases: key players in hormone signaling leading to stress-induced phenotypic plasticity. Int. J. Mol. Sci. 22, 1–15. doi: 10.3390/ijms22105136 PMC8152075 34066274

[B15] Fraudentali I. Rodrigues-Pousada R. A. Tavladoraki P. Angelini R. Cona A. (2020b). Leaf-wounding long-distance signaling targets atCuAOβ Leading to root phenotypic plasticity. Plants (Basel Switzerland). 9, 1–15. doi: 10.3390/plants9020249 PMC7076439 32075218

[B16] Ghuge S. A. Carucci A. Rodrigues-Pousada R. A. Tisi A. Franchi S. Tavladoraki P.. (2015a). The apoplastic copper AMINE OXIDASE1 mediates jasmonic acid-induced protoxylem differentiation in arabidopsis roots. Plant Physiol. 168, 690–707. doi: 10.1104/pp.15.00121 25883242 PMC4453780

[B17] Ghuge S. A. Carucci A. Rodrigues-Pousada R. A. Tisi A. Franchi S. Tavladoraki P.. (2015b). The MeJA-inducible copper amine oxidase AtAO1 is expressed in xylem tissue and guard cells. Plant Signaling Behav. 10, e1073872. doi: 10.1080/15592324.2015.1073872 PMC4883905 26241131

[B18] Groß F. Rudolf E. E. Thiele B. Durner J. Astier J. (2017). Copper amine oxidase 8 regulates arginine-dependent nitric oxide production in Arabidopsis thaliana. J. Exp. Bot. 68, 2149–2162. doi: 10.1093/jxb/erx105 28383668 PMC5447880

[B19] Heniquez A. Meissonnier G. Visentin V. Prévot D. Carpéné C. (2003). High expression of semicarbazide-sensitive amine oxidase genes AOC2 and AOC3, but not the diamine oxidase gene AOC1 in human adipocytes. Inflammation Res. 52 Suppl 1, S74–S75. doi: 10.1007/s000110300061 12755418

[B20] Imamura Y. Kubota R. Wang Y. Asakawa S. Kudoh J. Mashima Y.. (1997). Human retina-specific amine oxidase (RAO): cDNA cloning, tissue expression, and chromosomal mapping. Genomics 40, 277–283. doi: 10.1006/geno.1996.4570 9119395

[B21] Jalkanen S. Karikoski M. Mercier N. Koskinen K. Henttinen T. Elima K.. (2007). The oxidase activity of vascular adhesion protein-1 (VAP-1) induces endothelial E- and P-selectins and leukocyte binding. Blood 110, 1864–1870. doi: 10.1182/blood-2007-01-069674 17548577

[B22] Kaitaniemi S. Elovaara H. Grön K. Kidron H. Liukkonen J. Salminen T.. (2009). The unique substrate specificity of human AOC2, a semicarbazide-sensitive amine oxidase. Cell. Mol. Life Sci.: CMLS. 66, 2743–2757. doi: 10.1007/s00018-009-0076-5 19588076 PMC11115939

[B23] Kivi E. Elima K. Aalto K. Nymalm Y. Auvinen K. Koivunen E.. (2009). Human Siglec-10 can bind to vascular adhesion protein-1 and serves as its substrate. Blood 114, 5385–5392. doi: 10.1182/blood-2009-04-219253 19861682 PMC2978503

[B24] Krzywinski M. Schein J. Birol I. Connors J. Gascoyne R. Horsman D.. (2009). Circos: an information aesthetic for comparative genomics. Genome Res. 19, 1639–1645. doi: 10.1101/gr.092759.109 19541911 PMC2752132

[B25] Kurkijärvi R. Adams D. H. Leino R. Möttönen T. Jalkanen S. Salmi M. (1998). Circulating form of human vascular adhesion protein-1 (VAP-1): increased serum levels in inflammatory liver diseases. J. Immunol. (Baltimore. Md.: 1950). 161, 1549–1157. 9686623

[B26] Letunic I. Bork P. (2024). Interactive Tree of Life (iTOL) v6: recent updates to the phylogenetic tree display and annotation tool. Nucleic Acids Res. 52, W78–W82. doi: 10.1093/nar/gkae268 PMC11223838 38613393

[B27] Letunic I. Khedkar S. Bork P. (2021). SMART: recent updates, new developments and status in 2020. Nucleic Acids Res. 49, D458–d460. doi: 10.1093/nar/gkaa937 33104802 PMC7778883

[B28] Lopes de Carvalho L. Bligt-Lindén E. Ramaiah A. Johnson M. S. Salminen T. A. (2019). Evolution and functional classification of mammalian copper amine oxidases. Mol. Phylogenet. Evol. 139, 106571. doi: 10.1016/j.ympev.2019.106571 31351182

[B29] Maintz L. Novak N. (2007). Histamine and histamine intolerance. Am. J. Clin. Nutr. 85, 1185–1196. doi: 10.1093/ajcn/85.5.1185 17490952

[B30] Maintz L. Schwarzer V. Bieber T. van der Ven K. Novak N. (2008). Effects of histamine and diamine oxidase activities on pregnancy: a critical review. Hum. Reprod. Update 14, 485–495. doi: 10.1093/humupd/dmn014 18499706

[B31] Marchler-Bauer A. Derbyshire M. K. Gonzales N. R. Lu S. Chitsaz F. Geer L. Y.. (2015). Hurwitz DI et al: CDD: NCBI’s conserved domain database. Nucleic Acids Res. 43, D222–D226. doi: 10.1093/nar/gku1221 25414356 PMC4383992

[B32] McGrath A. P. Hilmer K. M. Collyer C. A. Shepard E. M. Elmore B. O. Brown D. E.. (2009). Structure and inhibition of human diamine oxidase. Biochemistry 48, 9810–9822. doi: 10.1021/bi9014192 19764817 PMC2791411

[B33] Minh B. Q. Schmidt H. A. Chernomor O. Schrempf D. Woodhams M. D. von Haeseler A.. (2020). IQ-TREE 2: new models and efficient methods for phylogenetic inference in the genomic era. Mol. Biol. Evol. 37, 1530–1534. doi: 10.1093/molbev/msaa015 32011700 PMC7182206

[B34] Murakawa T. Baba S. Kawano Y. Hayashi H. Yano T. Kumasaka T.. (2019). In crystallo thermodynamic analysis of conformational change of the topaquinone cofactor in bacterial copper amine oxidase. Proc. Natl. Acad. Sci. United. States America 116, 135–140. doi: 10.1073/pnas.1811837116 PMC6320532 30563857

[B35] Murray-Stewart T. R. Woster P. M. Casero R. A. Jr. (2016). Targeting polyamine metabolism for cancer therapy and prevention. Biochem. J. 473, 2937–2953. doi: 10.1042/BCJ20160383 27679855 PMC5711482

[B36] Peng W. Li W. Song N. Tang Z. Liu J. Wang Y.. (2021). Genome-wide characterization, evolution, and expression profile analysis of GATA transcription factors in brachypodium distachyon. Int. J. Mol. Sci. 22, 1–14. doi: 10.3390/ijms22042026 PMC7922913 33670757

[B37] Peng Y. Zhao K. Zheng R. Chen J. Zhu X. Xie K.. (2024). A comprehensive analysis of auxin response factor gene family in melastoma dodecandrum genome. Int. J. Mol. Sci. 25, 1–18. doi: 10.3390/ijms25020806 PMC10815038 38255880

[B38] Pietrangeli P. Nocera S. Mondovi B. Morpurgo L. (2003). Is the catalytic mechanism of bacteria, plant, and mammal copper-TPQ amine oxidases identical? Biochim. Biophys. Acta 1647, 152–156. doi: 10.1016/s1570-9639(03)00083-9 12686125

[B39] Planas-Portell J. Gallart M. Tiburcio A. F. Altabella T. (2013). Copper-containing amine oxidases contribute to terminal polyamine oxidation in peroxisomes and apoplast of Arabidopsis thaliana. BMC Plant Biol. 13, 109. doi: 10.1186/1471-2229-13-109 23915037 PMC3751259

[B40] Potter S. C. Luciani A. Eddy S. R. Park Y. Lopez R. Finn R. D. (2018). HMMER web server: 2018 update. Nucleic Acids Res. 46, W200–w204. doi: 10.1093/nar/gky448 29905871 PMC6030962

[B41] Qu Y. An Z. Zhuang B. Jing W. Zhang Q. Zhang W. (2014). Copper amine oxidase and phospholipase D act independently in abscisic acid (ABA)-induced stomatal closure in Vicia faba and Arabidopsis. J. Plant Res. 127, 533–544. doi: 10.1007/s10265-014-0633-3 24817219

[B42] Salmi M. Jalkanen S. (2019). Vascular adhesion protein-1: A cell surface amine oxidase in translation. Antioxid. Redox Signaling 30, 314–332. doi: 10.1089/ars.2017.7418 PMC6306676 29065711

[B43] Salmi M. Kalimo K. Jalkanen S. (1993). Induction and function of vascular adhesion protein-1 at sites of inflammation. J. Exp. Med. 178, 2255–2260. doi: 10.1084/jem.178.6.2255 8245796 PMC2191278

[B44] Salminen T. A. Smith D. J. Jalkanen S. Johnson M. S. (1998). Structural model of the catalytic domain of an enzyme with cell adhesion activity: human vascular adhesion protein-1 (HVAP-1) D4 domain is an amine oxidase. Protein Eng. 11, 1195–1204. doi: 10.1093/protein/11.12.1195 9930668

[B45] Schwelberger H. G. (2006). Origins of plasma amine oxidases in different mammalian species. Inflammation Res. 55 Suppl 1, S57–S58. doi: 10.1007/s00011-005-0041-1 16705381

[B46] Shukla V. Fatima T. Goyal R. K. Handa A. K. Mattoo A. K. (2020). Engineered ripening-specific accumulation of polyamines spermidine and spermine in tomato fruit upregulates clustered C/D box snoRNA gene transcripts in concert with ribosomal RNA biogenesis in the red ripe fruit. Plants (Basel Switzerland). 9, 1–20. doi: 10.3390/plants9121710 PMC7762058 33291784

[B47] Smith D. J. Salmi M. Bono P. Hellman J. Leu T. Jalkanen S. (1998). Cloning of vascular adhesion protein 1 reveals a novel multifunctional adhesion molecule. J. Exp. Med. 188, 17–27. doi: 10.1084/jem.188.1.17 9653080 PMC2525535

[B48] Tavladoraki P. Cona A. Angelini R. (2016). Copper-containing amine oxidases and FAD-dependent polyamine oxidases are key players in plant tissue differentiation and organ development. Front. Plant Sci. 7, 824. doi: 10.3389/fpls.2016.00824 27446096 PMC4923165

[B49] Tavladoraki P. Cona A. Federico R. Tempera G. Viceconte N. Saccoccio S.. (2012). Polyamine catabolism: target for antiproliferative therapies in animals and stress tolerance strategies in plants. Amino Acids 42, 411–426. doi: 10.1007/s00726-011-1012-1 21874532

[B50] Upadhyay R. K. Shao J. Maul J. E. Schomberg H. Handa A. K. Roberts D. P.. (2024a). Unlocking the role of novel primary/di-amine oxidases in crop improvement: Tissue specificity leads to specific roles connected to abiotic stress, hormone responses and sensing nitrogen. J. Plant Physiol. 303, 154374. doi: 10.1016/j.jplph.2024.154374 39522457

[B51] Upadhyay R. K. Shao J. Roberts G. E. Mattoo A. K. (2024b). Comparative genomics and evidence for an unusual polyamine oxidation pathway in aquatic duckweed (Spirodela polyrhiza L.). Curr. Plant Biol. 39, 100359. doi: 10.1016/j.cpb.2024.100359

[B52] Wang W. Paschalidis K. Feng J. C. Song J. Liu J. H. (2019). Polyamine catabolism in plants: A universal process with diverse functions. Front. Plant Sci. 10, 561. doi: 10.3389/fpls.2019.00561 31134113 PMC6513885

[B53] Wang Y. Tang H. Debarry J. D. Tan X. Li J. Wang X.. (2012). MCScanX: a toolkit for detection and evolutionary analysis of gene synteny and collinearity. Nucleic Acids Res. 40, e49. doi: 10.1093/nar/gkr1293 22217600 PMC3326336

[B54] Zarei A. Trobacher C. P. Cooke A. R. Meyers A. J. Hall J. C. Shelp B. J. (2015). Apple fruit copper amine oxidase isoforms: peroxisomal MdAO1 prefers diamines as substrates, whereas extracellular MdAO2 exclusively utilizes monoamines. Plant Cell Physiol. 56, 137–147. doi: 10.1093/pcp/pcu155 25378687

[B55] Zhang Z. B. Xiong T. Wang X. J. Chen Y. R. Wang J. L. Guo C. L.. (2024). Lineage-specific gene duplication and expansion of DUF1216 gene family in Brassicaceae. PloS One 19, e0302292. doi: 10.1371/journal.pone.0302292 38626181 PMC11020792

