# Supplementary figures and images for: The tree of life of copper-containing amine oxidases
Source: Front Plant Sci. 2025 Apr 24;16:1544527. doi: 10.3389/fpls.2025.1544527 (PMC12058724; doi:10.3389/fpls.2025.1544527)

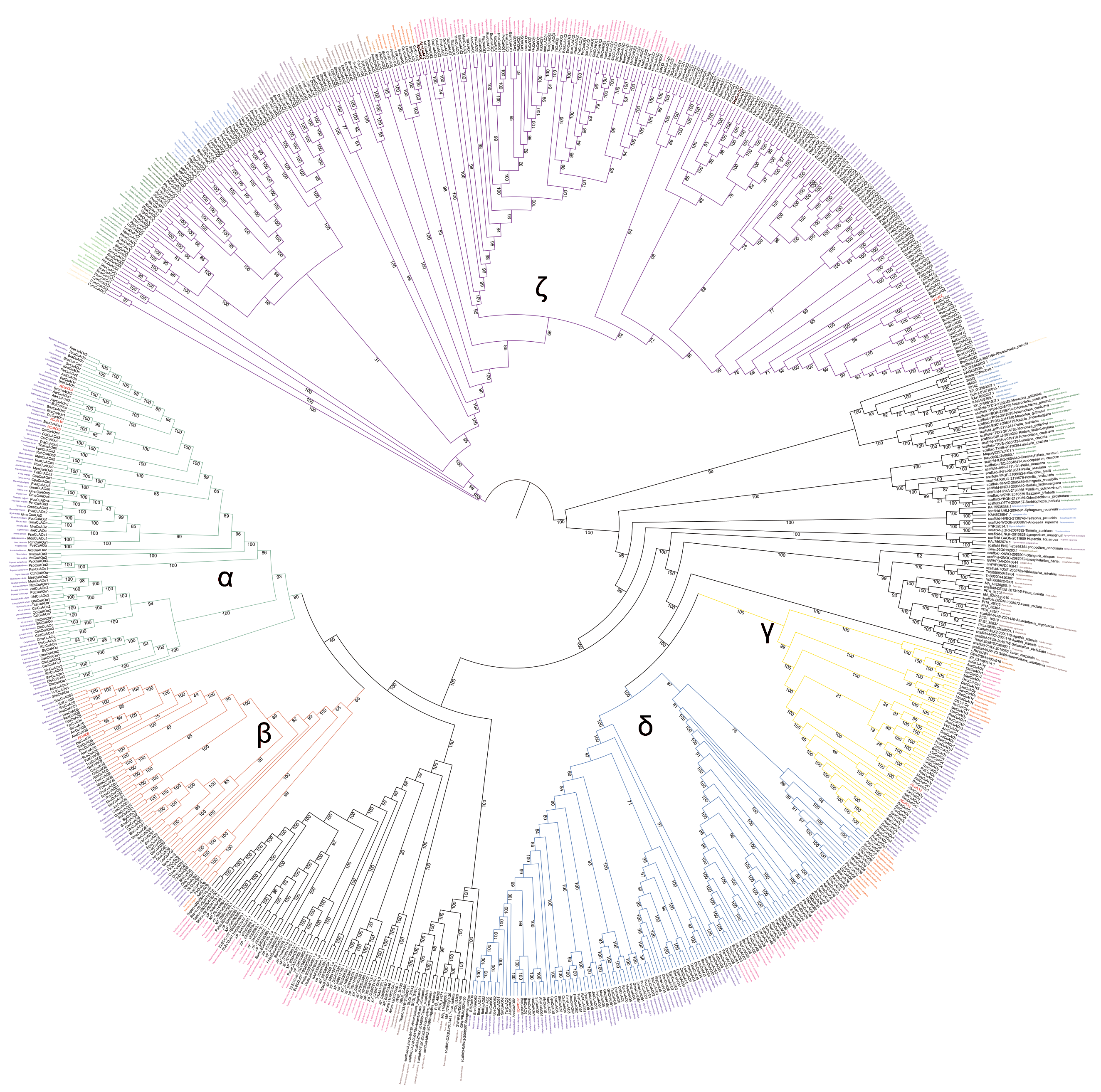

Supplement: Supplementary Figure 1 — Phylogenetic relationship of representative CuAO genes from plants. The phylogenetic tree was built using IQ-TREE2. Distinct branch colors represent various plant lineages. [file Image1.tiff]

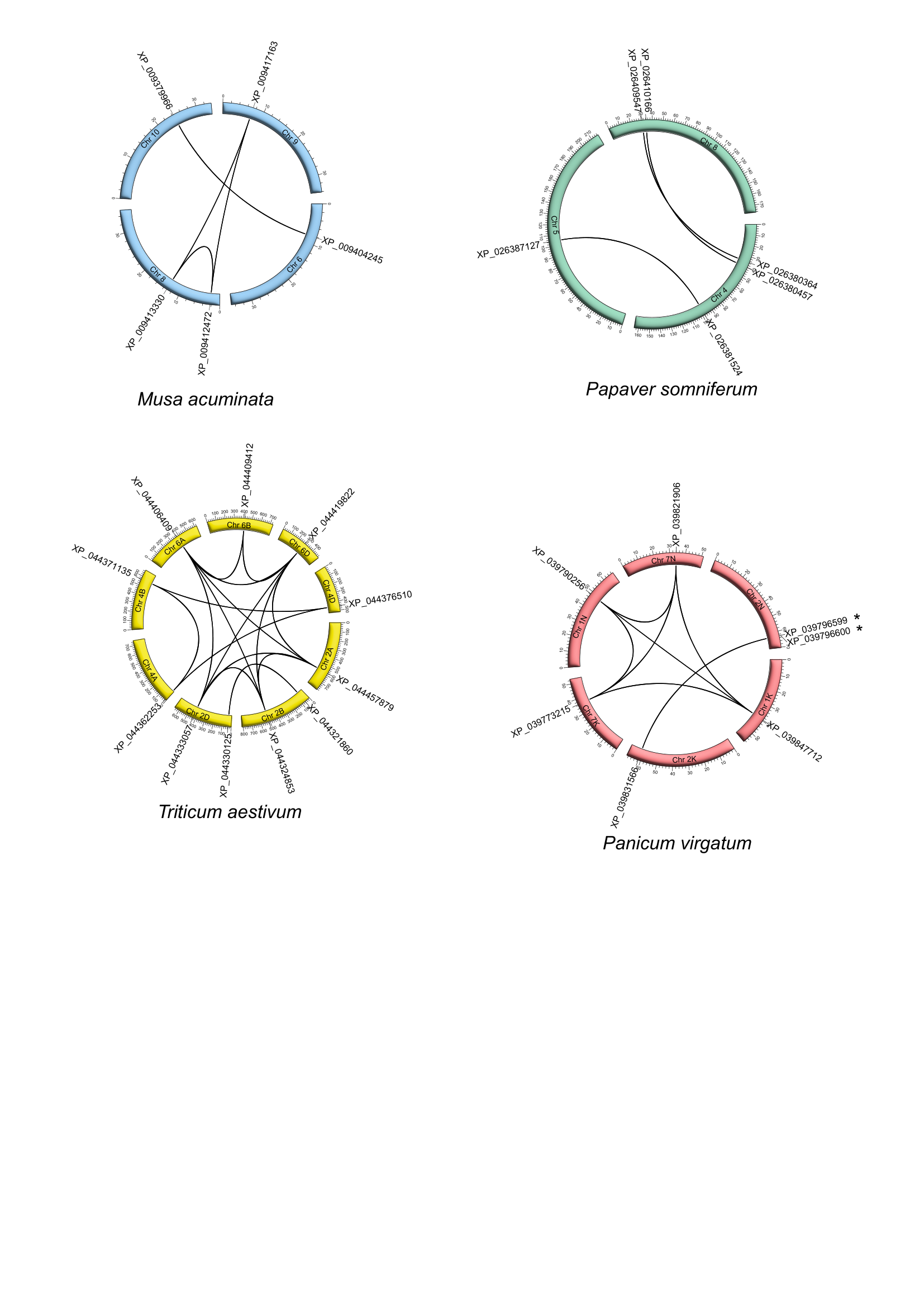

Supplement: Supplementary Figure 2 — Intraspecies syntenic relationships of CuAO genes in representative plants. The syntenic paralogs of CuAO genes are connected by black lines. The asterisk indicates tandem duplicate pairs. [file Image2.tiff]
